# Mechanism of megaloblastic anemia combined with hemolysis

**DOI:** 10.1080/21655979.2021.1952366

**Published:** 2021-09-19

**Authors:** Qiong Wu, Junru Liu, Xiaoxuan Xu, Beihui Huang, Dong Zheng, Juan Li

**Affiliations:** Department of Hematology, The First Affliated Hospital, Sun Yat-Sen University, Guangzhou, Guangdong, P.R. China

**Keywords:** Megaloblastic anemia, hemolysis, mean corpuscular volume, erythrocyte deformability, mechanical destruction

## Abstract

Megaloblastic anemia (MA) patients often exhibit hemolysis, but it is not clear whether there are other hemolytic mechanisms in addition to intramedullary hemolysis. We retrospectively analyzed the clinical characteristics of 124 MA patients, measured erythrocyte physical parameters in two patients with hemolysis and one healthy volunteer by atomic force microscopy, and measured 18F-FDG uptake in one MA patient with hemolysis. In multivariate analysis, hemolysis was associated with mean corpuscular volume (MCV) and indirect bilirubin. A receiver operating characteristic curve analysis, with sensitivity of 83.1% and specificity of 68.7%, suggested that the MCV cutoff value that predicts hemolysis is 116.4 fL. Hb was negatively correlated with MCV in the hemolysis group (r = −0.317, P = 0.007) but not in the nonhemolysis group. The erythrocyte peak-valley value, average cell surface roughness and surface area in the MA patients with hemolysis were significantly lower than those in controls (P < 0.05). 18F-FDG uptake by the liver and spleen was diffuse and increased in MA patients undergoing hemolysis. MA combined with extramedullary hemolysis could be caused by macrophages removing mechanically damaged erythrocytes and the retention of erythrocytes with decreased deformability when blood circulates through narrow spaces in the liver and spleen.

## GRAPHICAL ABSTRACT

**Figure:**
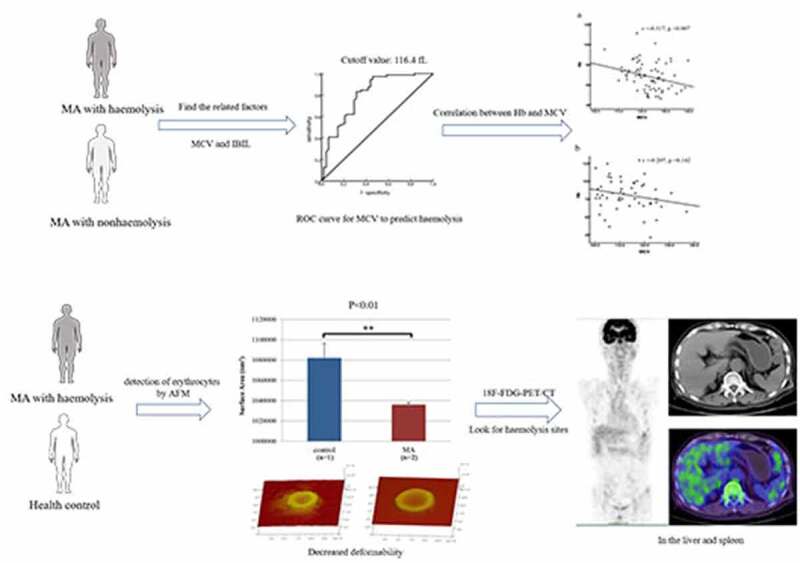

## 1. Introduction

Megaloblastic anemia (MA) accounts for approximately 3.6% of patients with anemia [1] and is mainly caused by the abnormal maturation of hematopoietic cells with disrupted DNA synthesis. Deficiencies in folic acid and vitamin B12, which are essential for DNA synthesis, are the main cause of MA. Cells in the bone marrow or other proliferative tissue are characterized by large and immature nuclei with chromatin, but the nucleus is surrounded by mature cytoplasm [2]. The asynchronous development of the nucleus and cytoplasm leads to cell apoptosis, abnormal erythropoiesis, intramedullary hemolysis, and typical abnormal cytomorphology in the blood and bone marrow [3], especially in erythron series.

However, in some MA patients, biochemical examination shows increases in the levels of LDH and TBIL, mainly IBIL, often occurring in patients with large MCV. These phenomena all suggest that MA patients could have excessive erythrocyte destruction. Some scholars have suggested that, before erythrocytes mature and are released into the peripheral blood, they can be destroyed in the bone marrow [4]. In vitro experiments have confirmed that the lack of folic acid or vitamin B12 in hematopoietic stem cells grown in cell medium can cause cell cycle arrest in S phase and chromosome breakage, resulting in cell apoptosis [5], and these apoptotic cells are eventually removed by phagocytes, leading to increased hemoglobin metabolite levels. Early research revealed that the lifespan of erythrocytes in MA patients is significantly shorter than that of normal erythrocytes [6]. The hemolysis in MA patients, in addition to intramedullary hemolysis, could be due to damage to dysplastic erythrocytes released into the blood circulation [7], but there is no associated literature to clarify this mechanism or the relationship between hemolysis and MCV.

According to our clinical observation and summary of MA patients with hemolysis, we believe that, in the process of peripheral blood circulation, the red blood cells of MA patients are destroyed due to the decreased deformability, and they cannot pass through the narrow gap, leading to hemolysis. Therefore, in this study, we retrospectively analyzed case data from MA patients to determine the cutoff value of MCV, to diagnose hemolysis and identify factors related to hemolysis and to determine the places where erythrocytes were destroyed in MA patients with hemolysis. This study was approved by the ICE for Clinical Research and Animal Trials of the First Affiliated Hospital of Sun Yat-sen University, with the following approval number: Lun Shen [2018] No. 201, date: 2018-8-29.

## 2. Materials and methods

### 2.1. Patients and their treatment

Anemia was defined as a hemoglobin (Hb) level <120 g/L in men and an Hb level <110 g/L in women with an Hb level <100 g/L in pregnant women. We retrospectively analyzed patients diagnosed with MA between January 2007 and November 2018 at the First Affiliated Hospital, Sun Yat-sen University. All patients had an MCV >100 fL in their routine blood work, and all of them were cured after treatment with vitamin B12 and folic acid. The exclusion criteria for patients in this study were: MA combined with liver or kidney function impairment or rheumatic diseases; presence of tumors; other diseases that affect MCV, such as iron deficiency anemia and thalassemia; anemia caused by other factors (blood loss, anemia associated with chronic disease, etc.); hemolysis caused by other factors (immune hemolytic anemia, drug-induced hemolysis, or artificial heart valve replacement); and treatment with drugs that interfere with DNA synthesis. Only 1 patient in this research received whole-body 18F-FDG PET/CT before treatment, and we measured the maximum standard uptake value (SUVmax) in the liver, spleen, bone marrow and mediastinum.

### 2.2. Erythrocyte physical parameter measurements

Two treatment-naïve patients diagnosed with MA combined with hemolysis and one healthy volunteer consented to a single erythrocyte measurement. A fasting venous blood sample (6 mL) was collected from each person in the morning. Heparin was used as an anticoagulant, and then the erythrocytes were separated by centrifugation, washed repeatedly, diluted, and finally fixed on a glass slide. The morphology and size of the fixed erythrocytes were measured in contact mode at room temperature. The gold-plated silica tip was used for atomic force microscopy (AFM, icon 3000, Bruker, USA) measurements. The tip diameter was 20 nm, and the elastic coefficient of the tip was 2.5 N/m. The samples were scanned by a 100 µm scanner. All of the images were analyzed with the IP2.1 software (Veeco) included with the instrument.

### 2.3. Statistical analysis

SPSS software, version 22.0, was used for statistical analysis. A nonparametric test was used to compare the patients’ baseline data. Univariate and multivariate logistic regression models were used to analyze the hemolysis-related factors. The discriminative ability of the MCV for hemolysis was analyzed using the area under the receiver operating characteristic (ROC) curve. The area under the ROC curve is presented with a 95% confidence interval (CI), and the Youden index was used to identify the maximal cutoff value. The t test was used to compare the erythrocyte parameters between the patients and healthy controls. All values were 2 sided, and P < 0.05 was considered statistically significant.

## 3. Results

In clinical work, we found that MA patients with hemolysis usually have a large MCV. We speculated that there might also be extramedullary hemolysis, not only hemolysis in situ. To this end, we retrospectively analyzed the clinical data of 124MA patients to determine the factors related to hemolysis. Atomic force microscopy was used to measure the physical parameters of the erythrocytes of MA patients with hemolysis to evaluate their deformability. Finally, the site of the destruction of red blood cells outside the medulla was found by PET-CT to confirm our hypothesis.

### 3.1. Baseline characteristics of the patients

A total of 124 patients were analyzed retrospectively in this research. The patients who exhibited increased LDH and TBIL, especially IBIL, levels and an elevated proportion or absolute value of reticulocytes in the peripheral blood were placed in the hemolytic group, while the other patients were placed in the nonhaemolytic group. There were 52 patients in the nonhaemolytic group and 72 in the hemolytic group. The majority of patients in the nonhaemolytic group had mild or moderate anemia, and in the hemolytic group, the majority had moderate or severe anemia. The MCV in the nonhaemolytic group was smaller than that in the hemolytic group (P < 0.05). As shown in Table 1, there were no significant differences in sex, age, or bone marrow puncture status between the two groups.

**Table 1.** Characteristics of patients

		Total		Nonhaemolysis		Hemolysis		P value
		n	%	n	%	n	%	
Sex								
	Male	65	52.42	22	42.31	43	59.72	0.055
	Female	59	47.58	30	57.69	29	40.28	
Age								
	≤60	45	36.29	22	42.31	23	31.94	0.495
	>60	79	63.71	30	57.69	49	68.06	
Bone marrow aspiration								
	Yes	59	47.58	21	40.38	38	52.78	0.23
	No	65	52.42	31	59.62	34	47.22	
Anemia								
	Mild	20	16.13	17	32.69	3	4.17	0.001
	Moderate	74	59.68	28	53.85	46	63.89	
	Severe	29	23.39	6	11.54	23	31.94	
	Very Severe	1	0.81	1	1.92	0	0.00	
MCV (fL)								
	100–110	23	18.55	21	40.38	2	2.78	<0.001
	110.1–120	48	38.71	21	40.38	27	37.50	
	120.1–130	41	33.06	8	15.38	33	45.83	
	≥130	12	9.68	2	3.85	10	13.89	

Mild anemia was defined as an Hb level >90 g/L, moderate anemia was defined as an Hb level <90 g/L and ≥ 60 g/L, severe anemia was defined as an Hb level <60 g/L and ≥ 30 g/L, and very severe anemia was defined as an Hb level <30 g/L.

Abbreviations: Hb, hemoglobin; MCV, mean corpuscular volume

### 3.2. Related factors of patients with hemolysis

There were no significant correlations of hemolysis with age, sex, serum folic acid or vitamin B12 content (P > 0.05). Hemolysis was found to be related to Hb (OR = 0.964, P = 0.002), MCV (OR = 1.162, P < 0.001), LDH (OR = 1.001, P = 0.013), TBIL (OR = 1.235, P < 0.001) and IBIL (OR = 1.300, P < 0.001) in the patients after univariate logistic regression analysis (Table 2). We found that hemolysis in the patients was only related to MCV (OR = 0.828, P = 0.008) and IBIL (OR = 0.822, P = 0.015) after multivariate logistic regression analysis (Table 3).

**Table 2.** Related factors of patients with hemolysis in univariate logistic regression analysis

	χ2	P value	OR	95% CI for OR
Hb	9.669	0.002	0.964	0.941–0.986
MCV	25.072	<0.001	1.162	1.096–1.233
LDH	6.211	0.013	1.001	1.000–1.001
TBIL	30.704	<0.001	1.235	1.146–1.331
IBIL	23.022	<0.001	1.300	1.168–1.447

Abbreviations: Hb, hemoglobin; MCV, mean corpuscular volume; LDH, lactate dehydrogenase; TBIL, total bilirubin; IBIL, indirect bilirubin; CI, confidence interval

**Table 3.** Related factors of patients with hemolysis in multivariate logistic regression analysis

	χ2	p value	OR	95% CI for OR
MCV	7.055	0.008	0.828	0.721–0.952
IBIL	5.926	0.015	0.822	0.702–0.962

Abbreviations: MCV, Mean Corpuscular Volume; IBIL, indirect bilirubin;

### 3.3. MCV cutoff value determination

**Figure 1.**
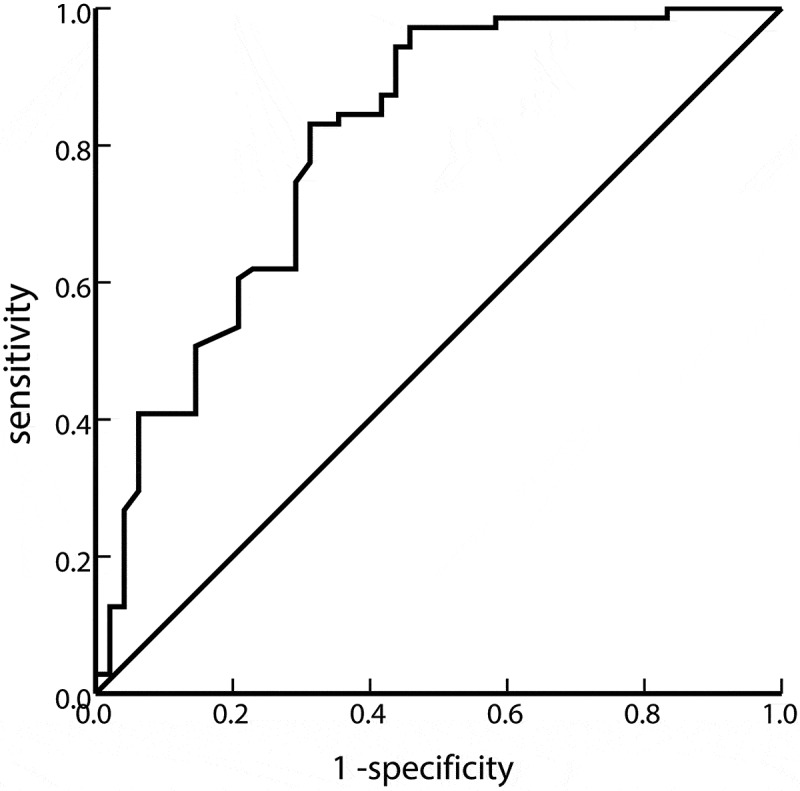
ROC curve between MCV and hemolysis

Since the IBIL level is affected by intramedullary hemolysis, we wanted to explore other hemolysis mechanisms; therefore, we used ROC curve analysis to find the best cutoff value for MCV to predict hemolysis. As shown in Figure 1, the MCV cutoff value was 116.4 fL. The sensitivity was 83.1%, the specificity was 68.7%, the area under the curve was 0.803, and the 95% CI was 0.719–0.887 (P < 0.001).

### 3.4. Correlation between Hb and MCV

**Figure 2.**
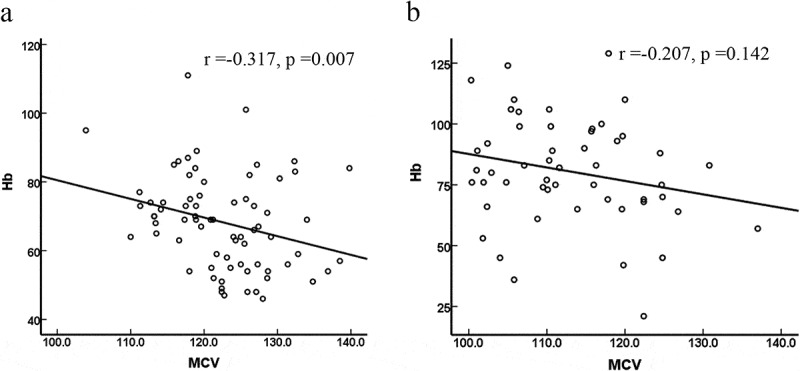
Correlation between Hb and MCV in two groups. a: MA patients with hemolysis; b: MA patients with nonhaemolysis

According to the above data analysis, we found that the larger that the MCV value is, the more likely that it is that MA will be combined with hemolysis. The correlations between Hb and MCV in the hemolytic group and the nonhaemolytic group were further analyzed. As shown in Figure 2, there was a negative correlation between Hb and MCV in only the hemolytic group (r = −0.317, P = 0.007); there was no significant correlation in the nonhaemolytic group (r = −0.207, P = 0.142).

### 3.5. Detection of erythrocytes by AFM

**Figure 3.**
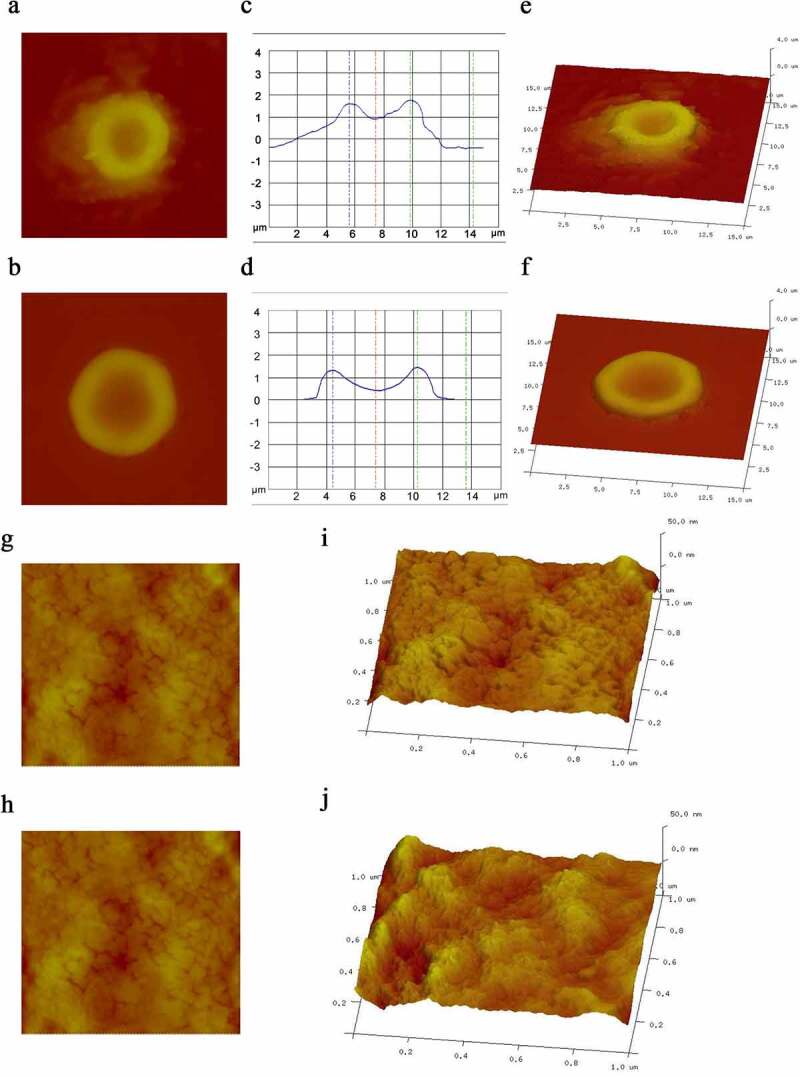
Erythrocyte morphology. a and b are images of a single erythrocyte in healthy controls and patients, respectively; c and d are altitude profiles of a single erythrocyte in healthy controls and patients, respectively; e and f are 3D models of the single erythrocyte in panels a and b, respectively; g and h are images of the surface membrane of a single erythrocyte in healthy controls and patients, respectively; i and j are the ultrastructures of the membrane of the erythrocyte in panels a and b, respectively

**Figure 4.**
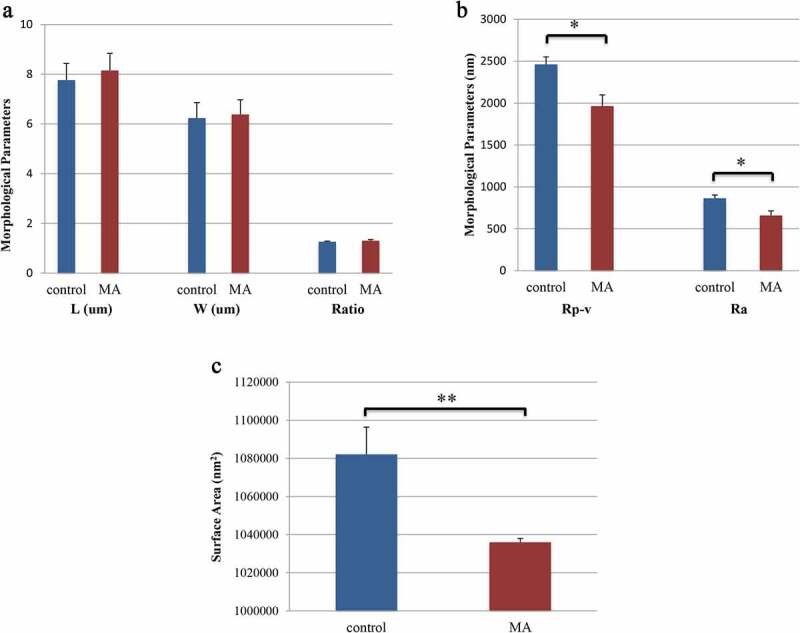
Erythrocyte physical parameters. a: The length of the erythrocytes in the control group was 7.76 ± 0.67 µm, and in the MA patients, it was 8.15 ± 0.69 µm (P = 0.722). The width of the erythrocytes in the control group was 6.24 ± 0.62 µm, and in the MA patients, it was 6.38 ± 0.59 µm (P = 0.878). The length-width ratio of the erythrocytes in the control was 1.26 ± 0.03, and in the MA patients, it was 1.31 ± 0.04 (P = 0.466). b: The Rp-v of the erythrocytes in the control group was 2462 ± 89.99 nm, and that in the MA patients was 1964 ± 134.50 nm (P = 0.021). The Ra of the erythrocytes in the control group was 865.44 ± 38.09 nm, and in the MA patients, it was 658.28 ± 55.23 nm (P = 0.020). c: The surface area of the erythrocytes in the control group was 1082161.56 ± 14204.96 nm^2^, and in the MA patients, it was 1036047.11 ± 1896.13 nm^2^ (P < 0.001). L: length of erythrocyte; W: width of erythrocyte; both of which were the maximum cell diameter. Rp-v: peak-valley value, which is the difference between the maximum and minimum height of the Z-axis on the cell surface in the analysis area; Ra: erythrocyte surface roughness, which is the mean roughness in the analysis area, namely the relief on the surface of the erythrocytes; * P value <0.05, ** P value <0.01

We measured erythrocyte physical parameter in two MA patients with hemolysis and one healthy volunteer by atomic force microscopy, whose characteristics were shown in Table 4. The erythrocytes from the patients were larger than those from the healthy controls, but the altitude of the erythrocytes from the patients was lower than that from the controls. We also obtained a 3D model of a single erythrocyte and images of the surface membrane of a single erythrocyte. It was clear that the Ra in the patients was significantly lower than that in the controls (Figure 3). The physical parameters, including the length, width, Rp-v and surface area of the erythrocytes, were also measured under a microscope (Figure 4). There were no significant differences in the length or width of the erythrocytes between the two groups, but the Rp-v and surface area of the erythrocytes from the patients were significantly lower than those of the erythrocytes from the control.

**Table 4.** Characteristics of healthy control and MA patients with hemolysis

	Gender	Age	Hb	MCV	TBIL	IBIL	LDH
Healthy control	Man	58	137	86	12.1	8.0	201
Patient 1	Man	54	83	118	45.0	31.6	292
Patient 2	Man	73	75	121	56.1	47.5	320

Abbreviations: Hb, hemoglobin (g/L); MCV, mean corpuscular volume (80–100 fL); TBIL, total bilirubin, (3.4–17.1 µmol/L); IBIL, indirect bilirubin (1.7–10.2 µmol/L); LDH, lactate dehydrogenase (135–215 U/L)

### 3.6. Liver and spleen SUVmax of a patient

**Figure 5.**
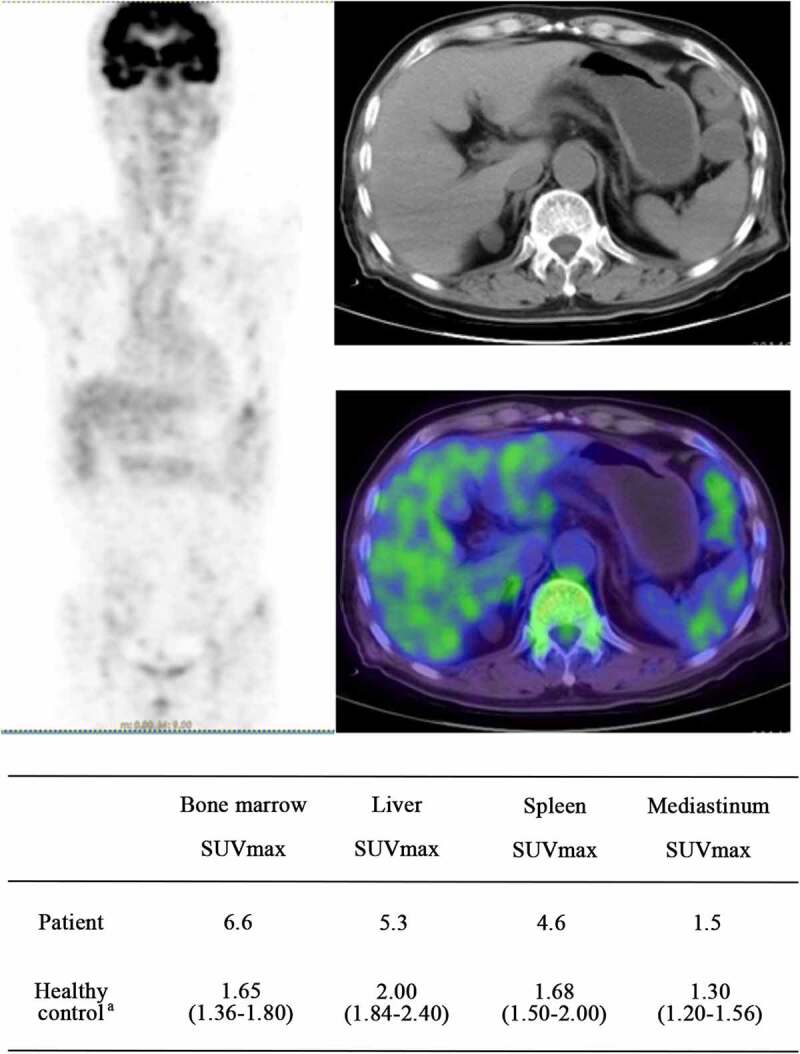
The images of whole body 18F-FDG-PET/CT. a:(median, quartile), SUVmax of healthy people who underwent 18F-FDG-PET/CT examination at our hospital; In the whole-body 18F-FDG-PET/CT images, the higher that the tissue uptake rate of FDG is, the higher that the SUV value is, and the darker that the color in the image is. As the SUV value rises, the image color changes from blue to green, yellow, or even red. Abbreviations: SUVmax, maximum standardized uptake value; 18F-FDG-PET/CT, 18F-fluorodeoxyglucose (18F-FDG) positron emission tomography and computed tomography (PET-CT)

A 74-year-old man diagnosed with MA combined with hemolysis received a whole-body 18 F-FDG-PET/CT scan before treatment. The patient’s clinical laboratory results were as follows: Hb 71 g/L, moderate anemia, MCV 119 fL, LDH 301U/L, TBil 63 µmol/L, and IBil 52 µmol/L. Diffuse 18F-FDG uptake significantly increased in the bone marrow, liver and spleen. The SUVmax values in the bone marrow, liver, and spleen were 6.6, 5.3, and 4.6, respectively, and these values were higher than those measured in healthy people who underwent whole-body 18F-FDG-PET/CT examination at our hospital [8] (Figure 5).

## 4. Discussion

Hemolysis in MA patients is mainly believed to be caused by the destruction of abnormal, immature, nucleated erythrocytes in the bone marrow before the cells can enter the peripheral blood circulation [7,9]. Some studies have suggested the possibility that extramedullary hemolysis occurs at the same time, but they have not clearly demonstrated the mechanism of extramedullary hemolysis. The results of the present study suggest that extramedullary hemolysis actually occurs in MA patients with hemolysis. In addition, extramedullary hemolysis is related to MCV in the peripheral blood. The larger that the MCV value is, the more likely that it is that MA will be associated with hemolysis.

Not all MA patients were associated with hemolysis. In this retrospective study, hemolysis was related to IBIL and MCV. If the hemolysis in a patient is only intramedullary hemolysis, only the level of IBIL, which is the metabolite of Hb after hemolysis, can be used to predict hemolysis, and the related factors will not include MCV, which is associated with erythrocytes in the peripheral blood. However, this relationship is not the case in MA patients. The incidence of hemolysis in MA patients is also related to MCV. It was also found that the life span of erythrocytes in the peripheral blood of MA patients was significantly shorter than that of erythrocytes from healthy people [6], further confirming the possibility of extramedullary hemolysis. In addition, we found that MCV was negatively correlated with Hb in the hemolysis group, and there was no correlation in the nonhaemolysis group. This finding revealed that extramedullary hemolysis in patients is related to the MCV value; the larger that the MCV is, the more likely that it is that hemolysis will occur, and the lower that the Hb level will be. The cutoff value of the MCV was 116.4 fL, which predicted hemolysis in MA patients well (area under the ROC curve was 0.803).

When blood circulates through constrictions, small capillaries, or splenic interendothelial slits, which are narrower and shorter than capillaries [10], erythrocytes must pass through apertures far smaller than their own cross-sectional size. Normal erythrocytes with good deformability can change shape to successfully pass through these narrow gaps [11]. In addition, the surface-to-volume ratio, cytoplasmic viscosity, and intrinsic membrane deformability regulate erythrocytes’ capacity to deform and transit narrow gaps [12].

The atomic force microscope, invented by Binig et al. in 1986, is a new instrument that can image and measure the surface of specimens at the atomic level [13]. Compared with traditional optical microscopy and scanning electron microscopy, AFM has the advantages of high spatial resolution, easy sample preparation, and a lack of invasion or destruction of the samples. Researchers can observe the ultrastructure of biological samples under physiological conditions and detect the mechanical properties of cells by AFM [14,15]. In this study, we used AFM to image the surface and measure the physical parameters of a single erythrocyte. The results showed that, compared with the parameters of the cells from the healthy controls, the Rp-v and Ra of the erythrocytes from the patients were obviously reduced, and these reductions led to reduced surface area. However, the MCV value of the MA patients was larger than that of the healthy controls, resulting in a significantly reduced ratio of surface area to volume. The decreased ratio led to reduced deformability. Therefore, we believe that the reduced deformability of the cells, indicating that they cannot withstand the shear stress of arterial circulation, prevents the maintenance of cell integrity during microcirculation and ultimately leads to hemolysis, and similar processes occur with erythrocytes in hereditary spherocytosis [16].

Abnormal and aged erythrocytes in the circulation are mainly removed by phagocytes in the spleen and liver [17,18]. When arterial blood within the splenic cord enters the sinus vein in the red pulp, erythrocytes must be squeezed into the space between the sinus endothelium and the stress fibers that extend underneath the basal plasma membrane, and they are arranged parallel to the cellular long axis [19]. If deformability decreases, erythrocytes cannot pass through the tiny gap; they will be destroyed and trapped in the spleen and then removed by macrophages. A similar process occurs in the liver.

Ogawa et al [20]. found that the SUVmax of arterial plaque detected by 18F-FDG PET/CT was positively correlated with the number and density of macrophages in the diseased vessel. Kawai et al. [21] also found that, in malaria-infected monkeys, the increased 18F-FDG uptake in the spleen was related to the activation of the spleen clearance system and the glucose consumption of the malaria parasite. These studies suggested that the activation of macrophages could lead to increased 18F-FDG uptake.

In this study, the SUVmax in the liver and spleen of the patients with MA combined with hemolysis showed diffuse increases by PET/CT, and the 18F-FDG uptake in the spleen and liver of our patient was significantly higher than that in normal people who underwent PET/CT at our hospital and people examined at another institution (liver SUVmax was 3.2 ± 0.8; spleen SUVmax was 2.4 ± 0.6 in healthy men) [22]. Therefore, we believe that erythrocytes with decreased deformability cannot pass through the narrow gaps in the liver and spleen as blood circulates through the body. In this study, the mechanical destruction and retention of erythrocytes were increased, and then macrophages in the liver and spleen were activated and gathered to remove these erythrocytes, ultimately leading to a significant increase in 18F-FDG uptake in the liver and spleen. Thus, we believe that the main sites of erythrocyte destruction in MA patients with extramedullary hemolysis are the liver and spleen.

However, there are still some limitations to this study. In this retrospective study, only one patient with hemolysis underwent PET/CT examination, and a larger sample size is needed. It is also necessary to further observe and compare 18F-FDG uptake in the spleen and liver of MA patients who do not exhibit hemolysis. In addition, we believe that there are a large number of activated macrophages in the liver and spleen of patients with hemolysis that remove mechanically destroyed and detained erythrocytes, but this hypothesis has not been confirmed by pathological results.

## 5. Conclusion

In conclusion, extramedullary hemolysis was found in patients diagnosed with MA combined with hemolysis. The larger that the peripheral blood MCV was, the more likely that it was that the patient had MA combined with hemolysis. Extramedullary hemolysis is mainly caused by mechanically damaging and retaining erythrocytes with significantly decreased deformability that cannot pass through narrow spaces during blood circulation.

## Research highlights

- We found the MCV cut-off value of MA patients with hemolysis.
- Atomic force microscopy was used to analyse and compare the erythrocyte parameters of MA patients with hemolysis and healthy controls.
- We revealed a new mechanism of hemolysis in MA patients.

## References

[1] Kaur N, Nair V, Sharma S, et al. A descriptive study of clinico-hematological profile of megaloblastic anemia in a tertiary care hospital. Med J Armed Forces India. 2018;74:365–370. 30449923 10.1016/j.mjafi.2017.11.005 PMC6224687

[2] Das KC, Das M, Mohanty D, et al. Megaloblastosis: from morphos to molecules. Med Princ Pract. 2005;14(Suppl 1):2–14. . 16103708 10.1159/000086179

[3] Khanduri U, Sharma A. Megaloblastic anaemia: prevalence and causative factors. Natl Med J India. 2007;20:172–175. 18085121

[4] Coleman DH, Donohue DM, Finch, et al. Erythrokinetics in pernicious anemia. Blood. 1956;11:807–820. 13355891

[5] Koury MJ, Price JO, Hicks GG. Apoptosis in megaloblastic anemia occurs during DNA synthesis by a p53-independent, nucleoside-reversible mechanism. Blood. 2000;96:3249–3255. 11050010

[6] Singer K, JC King, Robin S. The life span of the megalocyte and the hemolytic syndrome of pernicious anemia. J Lab Clin Med. 1948;33:1068–1078. 18880905

[7] London IM, West R. The formation of bile pigment in pernicious anemia. J Biol Chem. 1950;184:359–364. 15422004

[8] Chen M, Lu L, Li J, et al. Value of systemic PET/CT in the diagnosis and differential diagnosis of aplastic anemia. Oncol Lett. 2018;16:3215–3222. 30127917 10.3892/ol.2018.9049 PMC6096074

[9] Anderssen N. The activity of lactic dehydrogenase in megaloblastic anaemia. Scand J Haematol. 1964;1:212–219. 14214900 10.1111/j.1600-0609.1964.tb00017.x

[10] Deplaine G, Safeukui I, Jeddi F, et al. The sensing of poorly deformable red blood cells by the human spleen can be mimicked in vitro. Blood. 2011;117:e88–95. 21163923 10.1182/blood-2010-10-312801 PMC3062417

[11] Danielczok JG, Terriac E, Hertz L, et al. Red blood cell passage of small capillaries is associated with transient Ca(2+)-mediated adaptations. Front Physiol. 2017;8:979. 29259557 10.3389/fphys.2017.00979 PMC5723316

[12] Mohandas N, Phillips WM, Bessis M. Red blood cell deformability and hemolytic anemias. Semin Hematol. 1979;16:95–114. 384522

[13] Trache A, Meininger GA. Atomic force microscopy (AFM). Curr Protoc Microbiol. 2008;Chapter 2:Unit 2C.2. 10.1002/9780471729259.mc02c02s8 18770536

[14] Parot P, Dufrêne YF, Hinterdorfer P, et al. Past, present and future of atomic force microscopy in life sciences and medicine. J Mol Recognit. 2007;20:418–431. 18080995 10.1002/jmr.857

[15] Allison DP, Mortensen NP, Sullivan CJ, et al. Atomic force microscopy of biological samples. Wiley Interdiscip Rev Nanomed Nanobiotechnol. 2010;2:618–634. 20672388 10.1002/wnan.104

[16] Perrotta S, Gallagher PG, Mohandas N. Hereditary spherocytosis. Lancet. 2008;372:1411–1426. 18940465 10.1016/S0140-6736(08)61588-3

[17] Mebius RE, Kraal G. Structure and function of the spleen. Nat Rev Immunol. 2005;5:606–616. 16056254 10.1038/nri1669

[18] Theurl I, Hilgendorf I, Nairz M, et al. On-demand erythrocyte disposal and iron recycling requires transient macrophages in the liver. Nat Med. 2016;22:945–951. 27428900 10.1038/nm.4146 PMC4957133

[19] Drenckhahn D, Wagner J. Stress fibers in the splenic sinus endothelium in situ: molecular structure, relationship to the extracellular matrix, and contractility. J Cell Biol. 1986;102:1738–1747. 3084499 10.1083/jcb.102.5.1738 PMC2114233

[20] Ogawa M, Ishino S, Mukai T, et al. (18)F-FDG accumulation in atherosclerotic plaques: immunohistochemical and PET imaging study. J Nucl Med. 2004;45:1245–1250. 15235073

[21] Kawai S, Matsumoto J, Yamaguchi H, et al. Enhancement of splenic glucose metabolism during acute malarial infection: correlation of findings of FDG-PET imaging with pathological changes in a primate model of severe human malaria. Am J Trop Med Hyg. 2006;74:353–360. 16525091

[22] Zincirkeser S, Sahin E, Halac M, et al. Standardized uptake values of normal organs on 18F-fluorodeoxyglucose positron emission tomography and computed tomography imaging. J Int Med Res. 2007;35:231–236. 17542410 10.1177/147323000703500207

